# Molecular markers reliably predict post-harvest deterioration of fresh-cut lettuce in modified atmosphere packaging

**DOI:** 10.1038/s41438-018-0022-5

**Published:** 2018-04-01

**Authors:** Ivan Simko, Ryan J. Hayes, Maria-Jose Truco, Richard W. Michelmore, Rudie Antonise, Mark Massoudi

**Affiliations:** 1 U.S. Department of Agriculture, Agricultural Research Service, U.S. Agricultural Research Station, Crop Improvement and Protection Research Unit, 1636 East Alisal Street, Salinas, CA 93905 USA; 2 U.S. Department of Agriculture, National Forage Seed Production Research Center, 3450 SW Campus Way, Corvallis, OR 97331 USA; 3 The Genome Center and Department of Plant Sciences, University of California, Davis, CA 95616 USA; 4 KeyGene N.V., P.O. Box 216, 6700 AE Wageningen, The Netherlands; 5 Ag-Biotech, 9701 Blue Larkspur Lane, Suite A, Monterey, CA 93940 USA

## Abstract

Fresh-cut lettuce is popular, but highly perishable product. Genetic studies of two bi-parental populations derived from crossing parents with rapid and slow rates of deterioration showed that the deterioration rate is a heritable trait (broad spectrum heritability, *H*^2^ of 0.56–0.87). The major genetic determinant of the deterioration rate in both populations was the quantitative trait locus (QTL), *qSL4*, located on linkage group 4. This QTL explained 40–74% of the total phenotypic variation of the trait in the two populations. Saturating the *qSL4* region with single-nucleotide (SNP) markers allowed detection of six haplotypes in a set of 16 lettuce accessions with different rates of deterioration. Three of the haplotypes were always associated with very rapid rates of deterioration, while the other three haplotypes were associated with slow rates of deterioration. Two SNPs located 53 bp apart were sufficient to separate the 16 accessions into two groups with different rates of deterioration. The accuracy of markers-trait association was subsequently tested on 350 plants from seven F_2_ families that originated from crossing parents with different rates of deterioration. The *H*^2^ of deterioration rate in these seven families ranged from 0.64 to 0.90. The SNP-based analysis accurately identified individuals with rapid, intermediate, and slow rates of deterioration in each family. Intermediate rate of deterioration was found in individuals having heterozygous alleles at *qSL4*, indicating an additive effect of the alleles. The assay can be used for fast, accurate, and reliable identification of deterioration rate after processing for salad.

## Salad crops: Longer-lasting lettuce

Genetic studies have shown that the rate of deterioration of cut lettuce leaves in pre-packaged salads is a highly heritable trait, governed by gene regions that could be used to breed longer-lasting varieties. Many genetic studies have aimed at breeding better varieties of lettuce (Lactuca sativa), but most have focused upon those grown for whole heads, rather than the cut leaves that are becoming increasingly popular with consumers. An international team led by Ivan Simko, of the USDA in Salinas, California, have developed a genetic assay to distinguish fast- from slow-deteriorating lettuce varieties based on a single DNA region identified in a previous study. Their marker-based test may be useful in developing lettuces that show both disease resistance during cultivation, and a longer shelf life once leaves are cut for sale.

## Introduction

Lettuce (*Lactuca sativa* L.) is a popular vegetable predominantly grown for its leaves in moderate climates throughout the world^1^. Lettuce leaves are mainly consumed raw as cut leaves in salads, but are also used in sandwiches and side dishes. Since the introduction of modified atmosphere packaging (MAP), use of fresh-cut lettuce packaged in salad mixes has experienced a dramatic increase^2^ due to its convenience for consumers. Fresh-cut products usually involve harvesting whole, mature heads, cutting the leaves to a specified size, and then packaging salad in bags using modified atmospheres^3,4^. Since lettuce is highly perishable, MAP is designed to extend shelf-life while reducing the occurrence of wound-induced discoloration on cut surfaces^5^. Even so, deterioration of lettuce pieces seen as darkening, water logging, and decomposition may occur in MAP and make the product unmarketable. Lettuce cultivars, plant introductions, land races, and accessions differ in their rate of deterioration in a range of MAP schemes^6–8^. Genetic studies of a single bi-parental population showed that deterioration of fresh-cut lettuce in MAP is a heritable trait conditioned by a few quantitative trait loci (QTL)^9^. As a result, the rate of deterioration can potentially be manipulated through selective breeding.

Until recently, the cultivars used to make packaged salads have generally been bred for the same traits as those targeted in breeding cultivars for marketing as whole heads. Breeding lettuce cultivars specifically suited for fresh-cut processing could improve the efficiency of production and the quality of the product^10^. Most efforts directed toward selecting cultivars for use in fresh-cut processing involve characterizing existing cultivars or advanced selections prior to release for deterioration, browning, or other post-processing traits^8,11–14^. Limited work has been devoted for the development of tools for selection during early and intermediate generations. These generations are characterized by large and diverse populations with segregation within and between families. There are limited numbers of plants available for post-processing tests, which are destructive and require multiple heads for each data point. These generations need methods for trait selection that ideally are rapid, inexpensive, have a high heritability, and use limited amounts of plant material. Systems based on evaluation of individual leaves were developed to improve shelf‐life by selecting for leaf developmental and biophysical traits^15^. The method enables the use of tissue samples collected from plants, rather than destroying the entire plant to conduct a shelf‐life test. Early and promising results were found using automatization to accelerate the deterioration-screening process, making the testing of large populations more feasible^16^. In all these cases, plants still need to be grown in a field to harvest maturity, harvested, and processed. To shorten the time needed for development of new cultivars, breeders could move from selection based on phenotype to selection based on genotype. In this approach, molecular markers closely linked to the gene(s) of interest would be used to analyze DNA from a large number of individuals in the early stages of their development (or even seeds) without destroying the plants^17^. Such molecular marker-assisted selection (MAS) can improve the accuracy and reduce the time needed for the identification of desirable genotypes. The utility of genomic selection was demonstrated for selecting slow deteriorating lettuce inbred lines from a bi-parental cross, results that have applicability to single seed descent programs but are not directly applicable to early generations^18^.

We previously mapped QTLs for the rate of deterioration in fresh-cut lettuce stored in low oxygen MAP^9^, a storage condition typically used for fresh-cut lettuce in the United States. The three QTLs detected in the Salinas 88 (iceberg type) × La Brillante (Batavia type) mapping population were located on linkage groups (LG) 1, 4, and 9^9^. The QTL on LG4 (*qSL4*) always had the largest effect, was detected in all experiments, and accounted for 40–74% of the total phenotypic variation of the trait. Thus, *qSL4* is a suitable candidate for MAS, providing that the same QTL is involved in the deterioration process of other genotypes across multiple horticultural types of lettuce. This deterioration phenotype is predominantly caused by genetic determinants^9^ and is not substantially affected by concentrations of oxygen (O_2_) or carbon dioxide (CO_2_)^6^.

The objectives of the present study were (i) to analyze QTL(s) for the rate of deterioration in additional bi-parental mapping population, (ii) to develop an MAS assay based on the molecular markers linked to *qSL4*, and (iii) to test the accuracy of this MAS assay in segregating families that  originate from crosses between different horticultural types.

## Material and methods

### Workflow

**Fig. 1:**
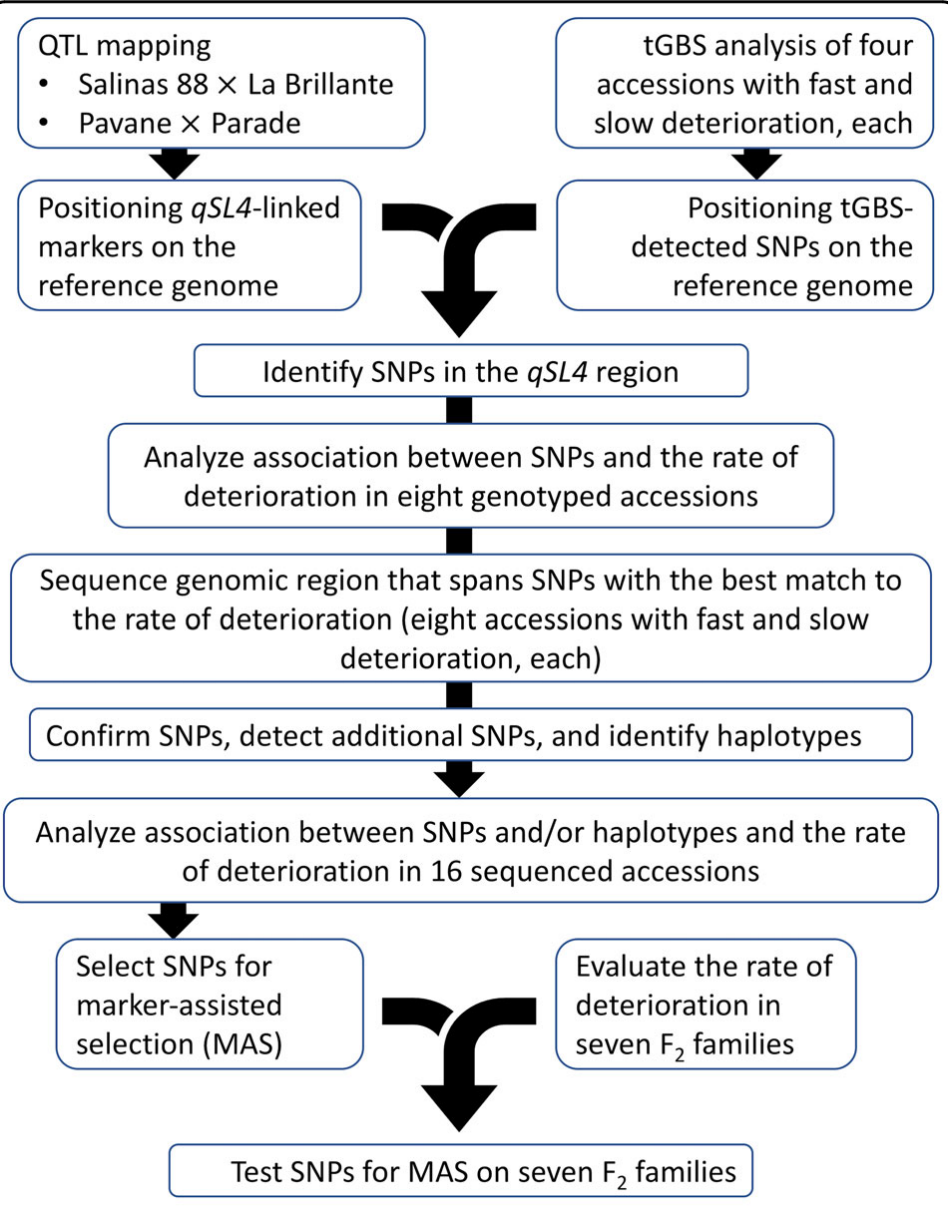
Workflow of the experiment

Linkage mapping of QTLs was performed on two mapping populations genotyped with molecular markers and phenotyped in multiple environments (Fig. 1). Molecular markers flanking the most significant QTL in each population were placed on the physical map of lettuce^19^. Because the chromosomal regions identified by these markers in the two different mapping populations overlapped, the region was saturated with additional molecular markers. These SNP markers were identified through tunable genotyping by sequencing (tGBS)^20^ on a set of eight accessions with different rates of deterioration. Marker alleles were compared to the phenotypes of the eight accessions to identify markers with the best match to the rate of deterioration. Chromosomal region around the markers with the best match to phenotypes was subsequently sequenced from 16 accessions originating from seven horticultural types of lettuce. Eight of these accessions previously showed a rapid deterioration after fresh-cut processing, while the other eight accessions have demonstrated an intermediate to slow rate of deterioration. Sequencing of these 16 accessions was performed to confirm existing SNPs, detect new SNPs, and identify distinct haplotypes. Based on the analyses of 16 accessions, SNPs and haplotypes with the best match to the rate of deterioration were identified. These SNPs were consequently used for genotyping of seven F_2_ families with individuals that showed differences in the rate of deterioration. Within each F_2_ family, a relationship between marker genotypes and phenotypes was evaluated. Detailed description of individual steps follows.

### Plant material

**Fig. 2:**
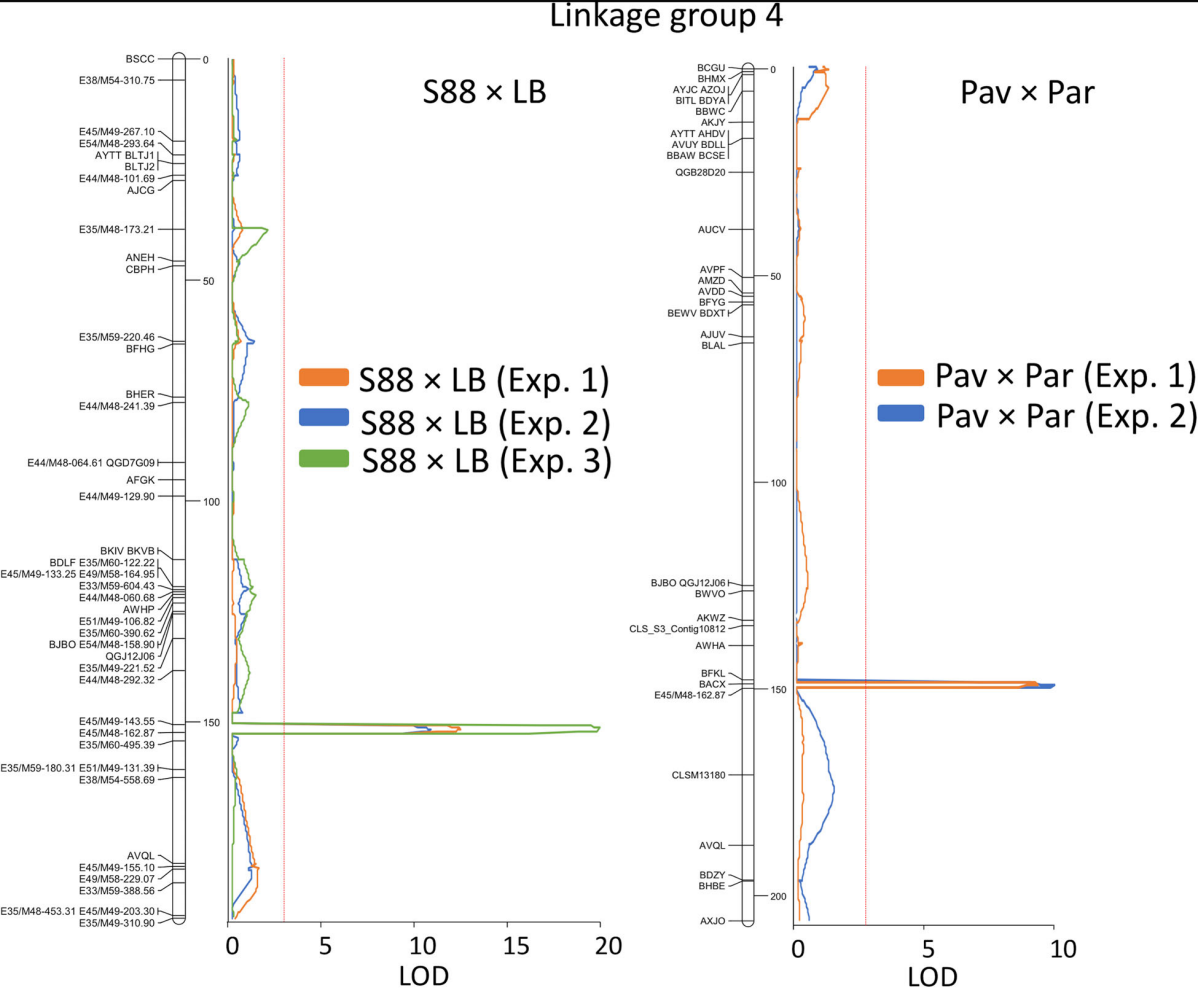
Position of the quantitative trait locus (QTL) for the rate of deterioration (*qSL4*) in fresh-cut lettuce located on linkage group 4. The panel on the left shows the position of the QTL previously detected in the Salinas 88 × La Brillante (S88 × LB) mapping population^9^. The panel on the right shows the position of the QTL currently detected in the Pavane × Parade (Pav × Par) mapping population. AFLP maker E45/M48-162.87 is closely linked to the QTL in both populations. The red lines parallel with the linkage maps show the significance threshold (*α* = 0.05). Distance in cM is indicated on the right side of the linkage map. LOD logarithm of odds. Note that only partially overlapping sets of molecular markers were used in two mapping populations, leading to somewhat different lengths of linkage maps

Linkage mapping was performed on two mapping populations: Salinas 88 × La Brillante (S88 × LB) and Pavane × Parade (Pav × Par). The S88 × LB population consisted of 95 recombinant inbred lines (RILs) in the F_7_ generation. Salinas 88 is a very slowly deteriorating iceberg cultivar, while Batavia-type cv. La Brillante always deteriorates rapidly (Table 1). This mapping population was genotyped with 104 SNP and 220 AFLP markers, and phenotyped after being grown in three environments (experiment 1 in 2010 in Yuma, Arizona; experiments 2 and 3 at different locations and growing seasons in 2010 in Salinas, California, Fig. 2.). A detailed description of this population and the results of QTL mapping were published previously^9^. The Pav × Par consisted of 78 RILs in the F_7_ generation. Butterhead cv. Parade consistently showed a slower rate of deterioration when processed for fresh-cut salad than the Latin-type cv. Pavane. This mapping population was genotyped with 257 SNPs. Molecular marker genotyping was conducted using the Illumina Golden Gate SNP assay (Illumina, San Diego, CA, USA). The linkage map was developed using JoinMap v. 2.0^21^. RILs plus both parents of this mapping population were grown in two field experiments, processed for salad, and evaluated for the rate of deterioration.

**Table 1:** Accessions used for studying the rate of deterioration in fresh-cut lettuce

Cultivar or plant introduction	Type of lettuce	Rate of deterioration^a^	Used in analyses			
			Linkage mapping	SNP-tGBS	Sequencing	F_2_ families
Balady Banha	Stem	Slow			x	x
Bandit	Romaine	Very rapid		x	x	
Bibb	Butterhead	Very rapid			x	x
Blonde Lente a Monter	Romaine	Very slow		x	x	x
Cobham Green	Butterhead	Rapid			x	
Eruption	Latin	Intermediate			x	x
King Henry	Romaine	Slow		x	x	x
La Brillante	Batavia	Very rapid	x	x	x	x
Little Gem	Latin	Very rapid		x	x	x
Lobjoits	Romaine	Slow		x	x	x
Parade	Butterhead	Slow	x			
Pavane	Latin	Very rapid	x		x	x
PI 491224	Romaine	Very rapid		x	x	x
Salinas	Iceberg	Very slow		x	x	x
Salinas 88	Iceberg	Very slow	x			
Tinto	Butterhead	Very rapid			x	
Two Star	Leaf	Slow			x	x
Valmaine	Romaine	Slow			x	

^a^Accessions with very slow, slow, and intermediate rates of deterioration are acceptable for fresh-cut processing, while those with rapid and very rapid rates of deterioration are unacceptable. Thus, all accessions with the acceptable rate of deterioration are called slow deteriorating (or having a slow rate of deterioration), while those with the unacceptable rate of deterioration are called rapidly deteriorating (or having a rapid rate of deterioration). The ratings were based on combined results from over 30 experiments performed during 10 years with more than 800 accessions.

Eight accessions (Bandit, Blonde Lente a Monter, King Henry, La Brillante, Little Gem, Lobjoits, PI 491224, and Salinas) were selected for tGBS analysis based on their rate of deterioration^7^. Four of these accessions regularly showed a slow or very slow rate of deterioration; while the other four accessions always deteriorated rapidly or very rapidly (Table 1). Similarly, out of 16 accessions selected for sequencing (Balady Banha, Bandit, Bibb, Blonde Lente a Monter, Cobham Green, Eruption, King Henry, La Brillante, Little Gem, Lobjoits, Pavane, PI 491224, Salinas, Tinto, Two Star, and Valmaine) eight were assessed as having a very slow, slow, or an intermediate rate of deterioration, and the other eight were assessed as having a rapid or very rapid rate of deterioration^7,13^ (Table 1). Accessions with very slow, slow, and intermediate rates of deterioration are described in the subsequent text as slow deteriorating (or having a slow rate of deterioration), while those with rapid and very rapid rates of deterioration are subsequently described as rapid deteriorating (or having a rapid rate of deterioration).

Seven F_2_ families were developed by mating pairs of lettuce accessions with distinct rates of deterioration: Lobjoits × PI 491224 (Lob × PI), Little Gem × Salinas (LG × Sal), La Brillante × Two Star (LB × TS), Pavane × Eruption (Pav × Eru), Bibb × King Henry (Bib × KH), Little Gem × Blonde Lente a Monter (LG x BLM), and Balady Banha × La Brillante (BB × LB) (Table 1). All F_2_ families and parental lines were grown in a single field experiment, processed for salad, and evaluated for deterioration.

### Field experiments and evaluations of deterioration

RILs and parents of the Pav × Par population were grown in two experiments located in Salinas, California in 2011 (experiment 1) and 2012 (experiment 2). Lettuce plants were planted and grown to maturity following standard agricultural procedures for the area, using raised beds of ~1 m wide and 25 cm high, with two parallel seed lines separated by 28 cm. The final distance between plants in a seed line was ~30 cm after thinning. RILs and parents were assigned into plots with ~15 plants per plot using a randomized complete block design (RCBD) with three replications. Six heads from each plot were harvested and placed in 4 °C forced-air cooled room for 1 day before processing. All the lettuce heads within a RIL or a parent were bulked within each field experiment and processed into cut lettuce^6,9^. After removing the cores, the leaves were cut into ~2.5 cm^2^ pieces with an Easy LettuceKutter (NEMCO, Hicksville, OH, USA) and thoroughly mixed. A sample size of 340 g was placed into a mesh bag, washed in 0.0016 mol l^–1^ NaOCL for 2 min, and dried in FP-35 food processing centrifuge (Bock Engineered Products, Toledo, OH, USA) at 2 × *g* for 5 min. In the next step, the samples were placed in transparent 22.8 × 30.5 cm bags made from 63.5-µm thick polyethylene coextruded film with an O_2_ transmission rating of 0.94 nmol s^–1^ m^–2^ Pa^–1^ (as determined by the manufacturer; Printpack, Atlanta, GA, USA). The bags of cut lettuce were triple-flushed with N_2_ before heat sealing. Each RIL or parent produced nine bags of cut lettuce. After processing, the bags were placed into carton boxes and stored in the dark at 4 °C.

Previously, we used the term ‘decay’ to describe the presence of water-soaked tissue eventually followed by tissue decomposition [6,9,10,13,14,16]. ‘Decay’, however, has been used to refer specifically to decomposition caused by microbial activity in other systems. Because the cause of the phenotype described in this work is unknown, we have revised our description to the more inclusive term ‘deterioration’. This terminology may also include, but is not restricted to, the loss of the tissue integrity caused by senescence or physiological conditions as well as microbial activity. Visual evaluations of deterioration were performed in weekly intervals, starting 1 week after processing and continuing until all bags with lettuce showed complete deterioration. Deterioration was recognized as the presence of water-soaked tissue. No other tissue blemishes, such as oxidative browning, pinking, or tipburn were considered in these evaluations. Evaluation of deterioration was performed on a 0 through 10 scale that corresponds to the estimated percentage of deteriorated tissue divided by 10 and rounded to the nearest whole number^7,9^. Weekly ratings were combined into the area under the deterioration progress stairs (AUDePS) values^22,23^ and used for statistical analyses. Visual ratings of deterioration correlate strongly with ion leakage, and detection of deterioration using chlorophyll fluorescence imaging and hyperspectral imaging^16^. Visual ratings performed by experienced raters have very high repeatability (intra-rater reliability), reproducibility (inter-rater reliability), and accuracy, thus making them convenient for rapid, non-destructive evaluations of deterioration in fresh-cut lettuce (Simko I and Hayes R, 2017, unpubl. data).

Plants from the seven F_2_ families and all parents of these families were grown in a single experiment in Salinas, California in 2014. Agricultural procedures were the same as described for the previous experiment, however, in this experiment ~100 plants per plot were grown in an unreplicated design. Fifty lettuce heads were harvested from each family, and 20 lettuce heads were harvested from each of the parental lines. Plants were processed as described for the Pav × Par population, with the exception of bulking. In this experiment, leaves from each head were cut, processed, stored, and evaluated for deterioration separately. Genomic DNA was extracted from fresh tissue of each head with the Qiagen DNeasy Plant Mini kit (Qiagen, Valencia, CA, USA).

### Linkage mapping and location of the markers on the physical map

QTL mapping on both populations (S88 × LB and Pav × Par) was performed with QGene v. 4.3.9 software^24^ using a scan interval of 1 cM and the single-trait, multiple interval mapping (SMIM) approach. The threshold for significant QTLs was set at the genome-wide *α* = 0.05 and determined through permutations with 1000 iterations^25^. The consensus chromosomal linkage map was created with MergeMap^26^. Molecular markers flanking the most significant QTL in each population were placed on the physical map of lettuce (version 8) assembled for cv. Salinas^19^.

### Genotyping by sequencing

The chromosomal region between markers flanking the most significant QTL was enriched with additional molecular markers. These SNP-based markers were identified on a set of eight accessions (Table 1 and Fig. 1) using tGBS (Data2Bio, Ames, IA, USA). Because all tested accessions were assumed to be homozygous, only markers with homozygous alleles detected in at least six out of the eight accessions were considered for further analyses. Positions of these markers were determined through alignment on the physical map of lettuce^19^.

### Sequencing

SNPs identified by the tGBS approach that were closely associated with deterioration phenotypes were further confirmed by additional sequencing (Fig. 1). The 269 bp long region around suggestive SNPs was amplified from 16 accessions (Table 1) using 2.5 × LightScanner Master Mix (BioFire Defense, Murray, UT, USA), 0.5 µM of each primer (P-012.1F: 5′- AGT GGT TAG TGG ATC GGG AGT -3′, P-012.1R: 5′- AGT TGA TCA CCT CCG CAA AC -3′), and 20 ng of genomic DNA. The master mix incorporates hot-start Taq polymerase, dNTPs, magnesium chloride, and LCGreen PLUS dye. Total of 10 µl of the PCR reaction mixture was added into individual wells of Hard-shell 96 thin well plate (BioRad, Hercules, CA, USA) and overlaid with 15 µl of mineral oil. The prepared PCR plate was centrifuged in an Eppendorf centrifuge 5810R (Eppendorf North America, Hauppauge, NY, USA) for 5 min at 2800 RPM. The PCR cycling conditions included denaturation of DNA template at 95 °C for 2 min, followed by 44 cycles of 95 °C for 30 s, 64 °C for 30 s, 72 °C for 30 s, and the final extension at 72 °C for 5 min. PCR products were cleaned using Sequencing clean-up kit (Zymo Research, Orange, CA, USA). Sequencing of amplicons from both directions was performed by Eton BioScience (San Diego, CA, USA). Sequence alignment and analyses were done using Geneious 10.1.3 (Auckland, New Zealand).

### Identification of alleles at SNP

SNPs associated with the rate of deterioration were analyzed by Kompetitive Allele Specific PCR (KASP) approach and high resolution DNA melting (HRM) approach. KASP analysis was performed by Ag-Biotech (Monterey, CA, USA). Amplification was performed using Veriti Thermal Cyclers (Applied Biosystems, Foster City, CA, USA), starting with 9 min at 94 °C, a touchdown phase of 10 cycles at 94 °C for 20 s and at 65 °C for 60 s with a 1 °C decrease in temperature per cycle, followed by 29 cycles of 94 °C for 20 s and 57 °C for 60 s. BMG FluoSTAR Omega (BMG Labtech, Cary, NC, USA) plate reader was used to read fluorescence signals for final SNP callings. PCR for HRM analysis was performed as described for sequencing, but using P-016F (5′-ACT TGG TAG TTA GGT GTG CGT-3′) and P-018R (5′-GTA GAC AGT GCC ACC CCA AC-3′) primers. To facilitate heteroduplex formation prior HRM analysis, samples were heated to 95 °C for 30 s, followed by cooling to 25 °C for 30 s. HRM analysis was conducted using LightScanner 96 Hi-Res Melting Systemset (formerly, Idaho Technology, Salt Lake City, UT, USA).

### Statistical analyses

Heritability for the rate of deterioration was calculated from AUDePS scores in two ways. In the Pav × Par population, each bag of lettuce was treated as a separate replicate^9,27^. Only data from RILs, but not parents, were used in the calculation: *H*^2^ = *σ*^2^_G_/(*σ*^2^_G_ + *σ*^2^_E_), where *σ*^2^_G_ and *σ*^2^_E_ are estimates of the variance component for genotype, and environment, respectively. In each of the seven F_2_ families, heritability was calculated from the AUDePS variances among plants within the two parents (*σ*^2^_P1_, *σ*^2^_P2_) and F_2_ families (*σ*^2^_F2_). In this approach, variances of both parents are considered to be caused by environmental effects only, while variances within each family are due to both genetic and environmental effects: *H*^2^ = [*σ*^2^_F2_ − (*σ*^2^_P1_ + *σ*^2^_P2_)/2]/*σ*^2^_F2_.

The mode of *qSL4* inheritance was estimated from mean values of AUDePS for SNPs linked to the gene. The *d/a* ratio was calculated from the values of dominance (*d*) and additivity (*a*), where *d* = *µ*_rs_ − (*µ*_rr_ + *µ*_ss_)/2, *a* = (*µ*_rr_ –* µ*_ss_)/2, and *µ*_rr_, *µ*_ss_, and *µ*_rs_ are the mean values of AUDePS for RILs homozygous for alleles linked to a rapid rate of deterioration, a slow rate of deterioration, or heterozygous.

Basic mathematical calculations were performed in Microsoft Excel v. 15.33 (Microsoft, Redmond, WA, USA), while statistical analyses were executed using JMP software v. 11.1.1 (SAS Institute, Cary, NC, USA).

## Results

**Fig. 3:**
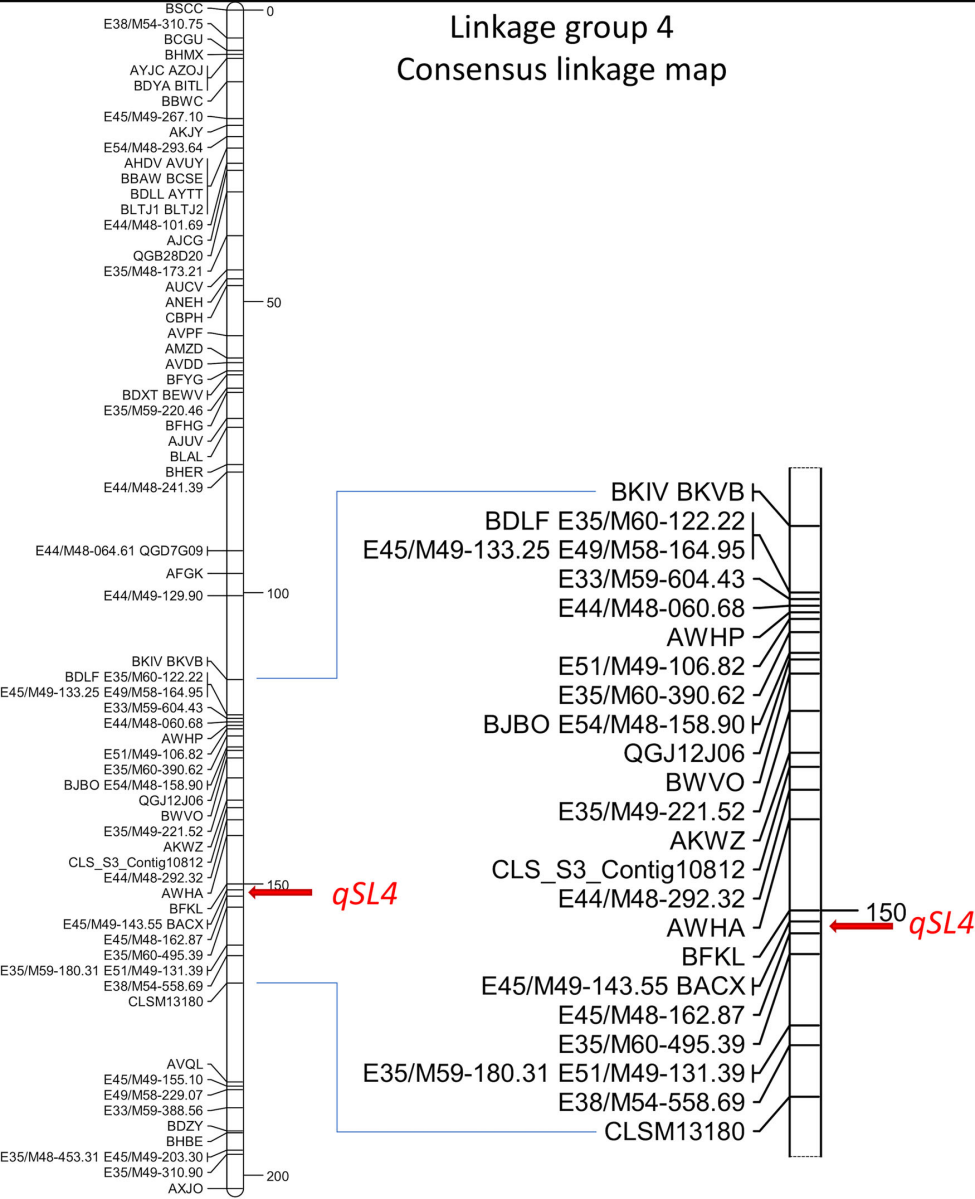
Position of *qSL4* on the consensus molecular map of linkage group 4. The consensus map was calculated from two maps developed for the Salinas 88 × La Brillante (S88 × LB) mapping population^9^ and the Pavane × Parade (Pav × Par) mapping population. The panel on the right shows details of the map with molecular markers in ~50 cM area around *qSL4*. Distance in cM is indicated on the right side of the linkage map

**Fig. 4:**
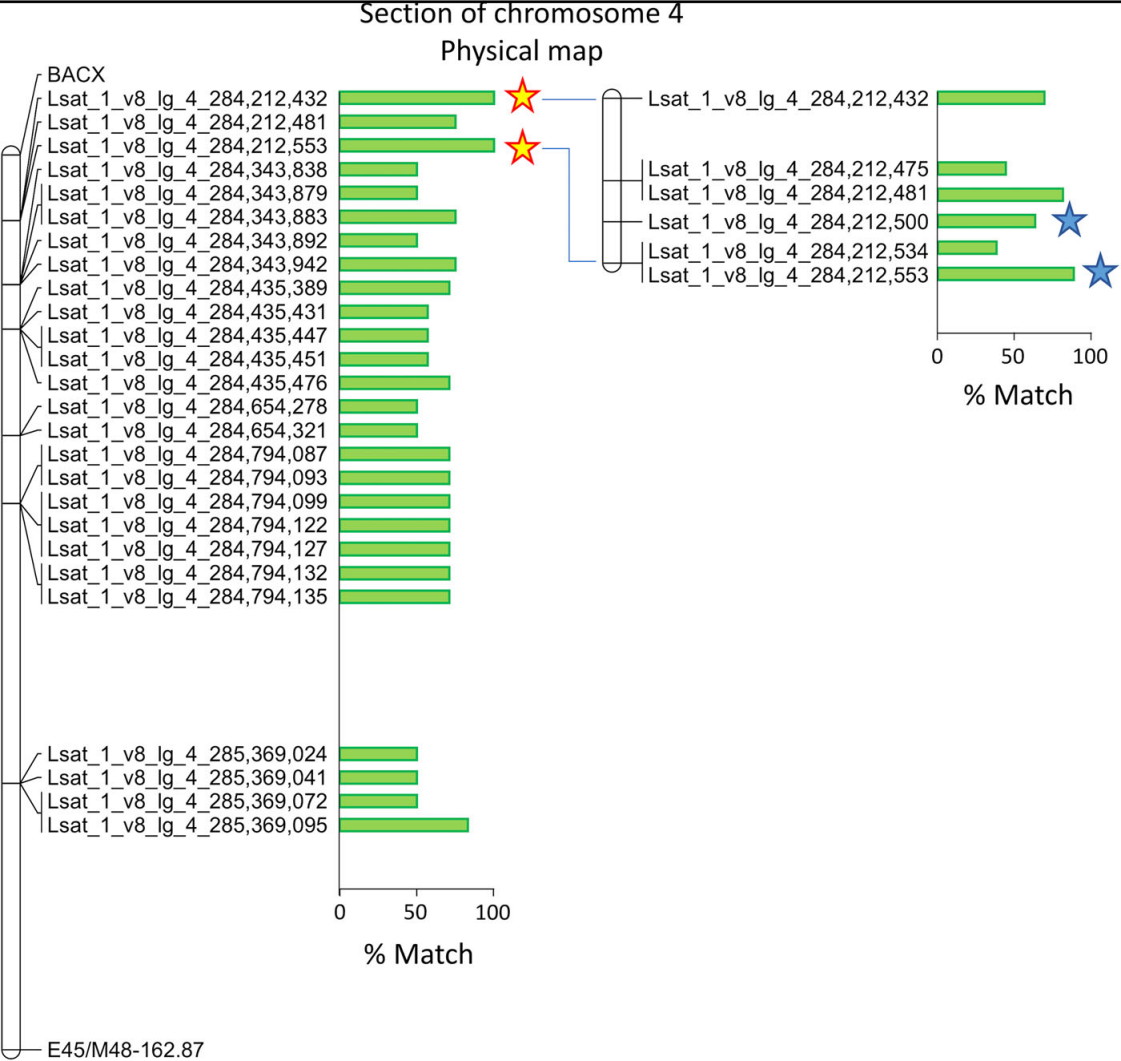
Position of SNP markers in the BACX-E45/M48-162.87 region of chromosome 4 and their association with the rate of deterioration in fresh-cut lettuce. The panel on the left shows the percentage of cultivars, where the 'slow' allele and 'rapid' allele of 26 SNPs matches the deterioration phenotype in eight accessions (four with a rapid and a slow rate of deterioration, each). The yellow/red stars indicate two SNPs with 100% match to the rate of deterioration. The genomic region spanning these two SNPs was selected for further analyses. The panel on the right shows the percentage of cultivars matching the projected alleles of six SNPs identified in this region through sequencing of 16 accessions (eight with a rapid and a slow rate of deterioration, each). These SNPs correspond to those highlighted in black in Fig. 5. The blue stars indicate two SNPs that can distinguish accessions with a rapid or a slow rate of deterioration. These two SNPs were used to design PCR-based assays for marker-assisted selection. The last nine numerals in the marker name indicate the position of the SNP on the physical map of lettuce^19^ version 8. The distance between midpoints of markers BACX and E45/M48-162.87 is 1,839,520 bp

The AUDePS scores that show the rate of deterioration were approximately normally distributed in all experiments performed with the Pav × Par mapping population and seven F_2_ families. In the first experiment with the Pav × Par population, the mean AUDePS scores for RILs was 626, while the mean values detected for cvs. Parade and Pavane were 575 and 698, respectively (Table 2). In the second experiment, the mean AUDePS values for RILs (411), cv. Parade (362), and cv. Pavane (460) were lower than in the first experiment. It is not possible to directly compare AUDePS scores across different experiments, because deterioration phenotypes were evaluated for a different number of weeks. In both experiments, however, cv. Parade showed a significantly (*p* < 0.001) slower rate of deterioration (lower AUDePS scores) than cv. Pavane. Broad sense heritability for the rate of deterioration was 0.62 (experiment 1) and 0.61 (experiment 2). Only a single, significant QTL was detected in each experiment. This QTL was flanked by markers BACX and E45/M48-162.87, and located approximately 149.2 to 149.5 cM from the top of LG4 (Fig. 2 and Table 2). The QTL effect was similar in both experiments, with the LOD scores of 9.82 and 9.23, and the total phenotypic variation explained by the QTL (R^2^%) at 44% and 42%, respectively. RILs carrying “Parade” alleles in this chromosomal region had significantly slower rates of deterioration compared to the RILs with “Pavane” alleles (Table 2, last two columns). Comparison of results from the Pav × Par population and the previously analyzed S88 × LB population^9^ indicates that the major QTL (*qSL4*) for deterioration is located in the same region of chromosome 4 in both populations (Fig. 2). The consensus linkage map was used to identify two makers, BACX and E45/M48-162.87, which flank the QTL (Fig. 3). This QTL was consistently detected in two mapping populations developed from different types of lettuce and tested in five different environments (Pav × Par in two environments, S88 × LB in in three environments). Alignment of the consensus linkage map (Fig. 3) and the physical map of the lettuce^19^ showed that the two markers flanking *qSL4* are separated by ~1.1 cM spanning 1.8 Mb (Figs. 3 and 4).

**Table 2:** Results of quantitative trail locus (QTL) mapping in the Parade × Pavane (Pav × Par) mapping population

Experiment	Parade AUDePS^a^	Pavane AUDePS^a^	RILs AUDePS^a^	*H* ^2^	QTL interval and location	LOD	*R*^2^%	Parade alleles^b^ AUDePS^a^	Pavane alleles^b^ AUDePS^a^
Pav × Par Exp. 1	575 (544–605)	698 (688–709)	626 (620–633)	0.62	BACX-E45/M48-162.87 149.5 cM	9.82	44	581 (562–600)	666 (651–680)
Pav × Par Exp. 2	362 (338–387)	460 (438–482)	411 (405–418)	0.61	BACX-E45/M48-162.87 149.2 cM	9.23	42	366 (347–386)	452 (435–470)

*H*^2^ broad sense heritability, *LOD* logarithm of odds, *R*^2^% percent of the phenotypic variation explained by the QTL

^a^Mean values of the area under the deterioration progress steps (AUDePS), and 95% confidence interval of the mean. Lower AUDePS score indicates a slower rate of deterioration.

^b^AUDePS values for recombinant inbred lines (RILs) with either “Parade” of “Pavane” alleles

To saturate the BACX to E45/M48-162.87 region with more molecular markers, we used tGBS to genotype eight accessions representing different horticultural types and rates of deterioration (Table 1). Genotyping identified 117 SNPs located between markers BACX and E45/M48-162.87. Ninety-one of these markers were detected in less than six accessions, or were in a heterozygous state; these were eliminated from further consideration. Alleles of the remaining 26 SNPs were compared to the phenotypes of the eight accessions. The match between the rate of deterioration (either rapid or slow) and SNPs alleles ranged from 50 to 100% (Fig. 4, left panel). The two SNPs (Lsat_1_v8_lg_4_284,212,432 and Lsat_1_v8_lg_4_284,212,553) with the best match to phenotypic data were separated by only 121 bp. An additional SNP (Lsat_1_v8_lg_4_284,212,481) with 75% match to the rate of deterioration was located between these two markers. Therefore, the chromosomal region containing these three SNPs was selected for further studies.

Sixteen accessions with slow and rapid rates of deterioration were chosen (Fig. 1 and Table 1, “Sequencing” column) to confirm selected SNPs, identify additional SNPs, and test SNP–phenotype associations. Sequencing of the 269 bp region from 16 accessions confirmed all three of the original SNPs and identified three additional SNPs (Lsat_1_v8_lg_4_284,212,475, Lsat_1_v8_lg_4_284,212,500, and Lsat_1_v8_lg_4_284,212,534) located in this amplicon. The test of association showed that no individual SNP had a perfect match to the known phenotypes, with the percentage of match ranging from 37.5% (Lsat_1_v8_lg_4_284,212,534) to 87.5% (Lsat_1_v8_lg_4_284,212,553) (Fig. 4, right panel).

**Fig. 5:**
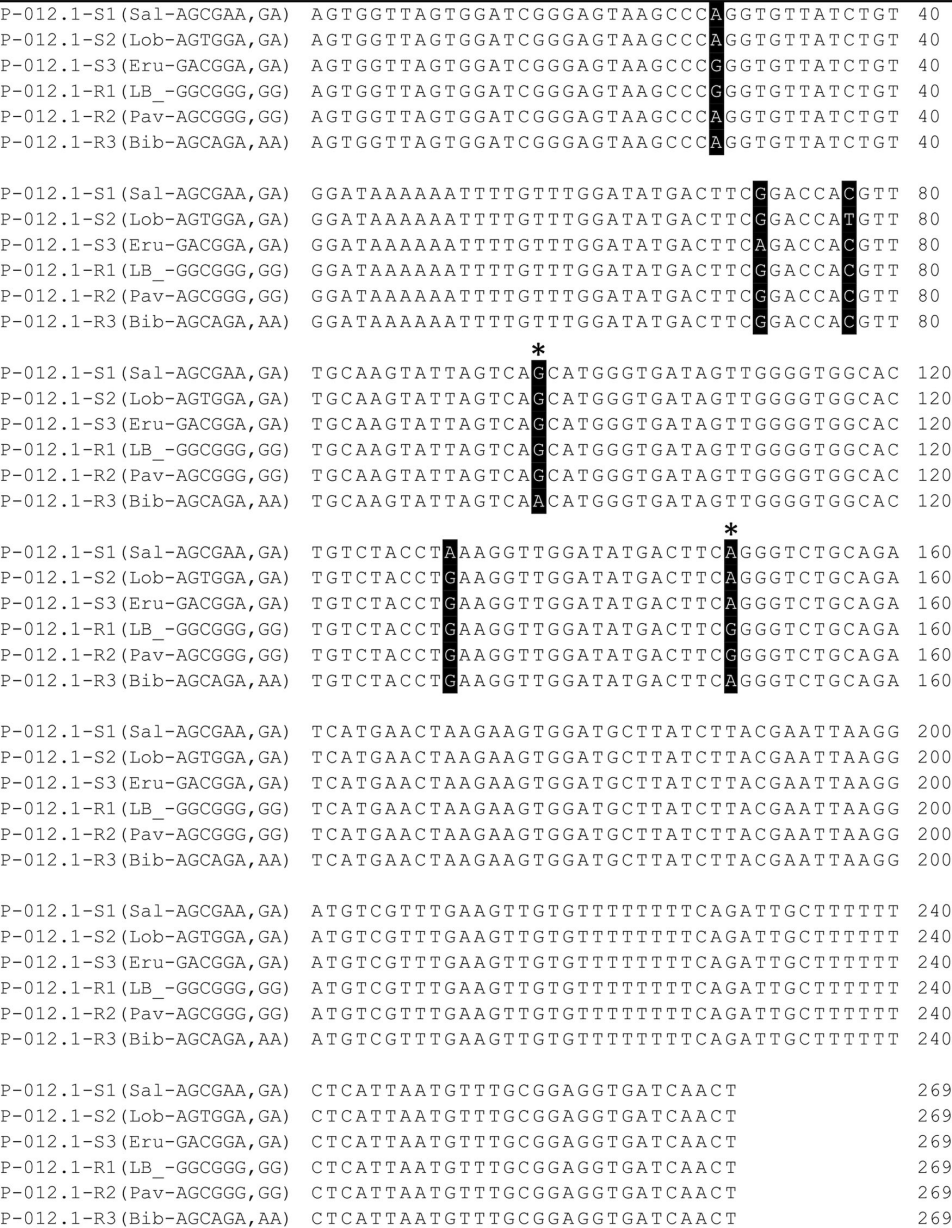
Sequence of P-012.1 amplicon and the positions of detected SNPs. Six SNPs identified at positions 28, 71, 77, 96, 130, and 149 are highlighted by black color. These six SNPs reveal six distinct haplotypes. Three of the haplotypes (P-012.1-S1, -S2, and –S3) are associated with a slow or an intermediate rate of deterioration, while the other three haplotypes (P-012.1-R1, -R2, and –R3) are associated with a rapid rate of deterioration in fresh-cut lettuce. Two SNPs at the positions 96 (P-012.1-096) and 149 (P-012.1-149) can be used to differentiate between accession with slow and rapid rates of deterioration. These two SNPs (indicated by asterisks) correspond to SNPs Lsat_1_v8_lg_4_284,212,500 and Lsat_1_v8_lg_4_284,212,553 on the physical map of lettuce genome^19^, version 8

The combination of six SNPs in the amplicon generated by the P-012.1 forward and reverse primers (hereafter referred to as the P-012.1 amplicon) revealed six haplotypes (Fig. 5). Haplotype P-012.1-S1 with alleles AGCGGA at the six SNP positions was detected in two cultivars (Blonde Lente a Monter and Salinas), haplotype P-012.1-S2 with alleles AGTGGA was detected in five cultivars (Balady Banha, King Henry, Lobjoits, Two Star, and Valmaine), and haplotype P-012.1-S3 with alleles GACGGA was detected in only cv. Eruption. All three of these haplotypes were associated with very slow to intermediate rates of deterioration. Three other haplotypes associated only with a rapid rate of deterioration were: P-012.1-R1 with alleles GGCGGG detected in four accessions (Bandit, La Brillante, Little Gem, and PI 491224), P-012.1-R2 with alleles AGCGGG detected in two cultivars (Cobham Green and Pavane), and P-012.1-R3 with alleles AGCAGA detected also in two cultivars (Bibb and Tinto).

Only two (Lsat_1_v8_lg_4_284,212,500 and Lsat_1_v8_lg_4_284,212,553) of the six SNPs were needed, however, to tag haplotypes associated with either a rapid or a slow rate of deterioration. For simplicity, these two SNPs were renamed based on their position at the P-012.1 amplicon as P-012.1-096 (original Lsat_1_v8_lg_4_284,212,500) and P-012.1-149 (original Lsat_1_v8_lg_4_284,212,553). The alleles of P-012.1-149 can be used to distinguish between accessions having slow (allele “A”) or rapid (allele “G”) rates of deterioration, with the exception of cultivars carrying the P-012.1-R3 haplotypes. To identify the P-012.1-R3 haplotype, alleles at the SNP P-012.1-096 need to be analyzed as well (allele “A“ indicates P-012.1-R3 haplotype, allele “G” indicates any other haplotype) (Fig. 5). The “GA” allele combination at these two SNPs was consistently associated with a slow rate of deterioration, while the “GG” or “AA” combination of alleles was always associated with a rapid rate of deterioration. The “AG” allele combination was never detected. The two SNPs (P-012.1-096 and P-012.1-149) tagging haplotypes associated with a rapid or a slow rate of deterioration were subsequently tested for their predictive accuracy in seven F_2_ families.

The mean AUDePS of the parents used to develop the F_2_ families ranged from 84 in cv. Lobjoits to 446 in cv. Pavane (Table 3). A significant difference (*p* < 0.001) for AUDePS was detected between the parents of each family. Deterioration variances among individuals in each family was significantly greater (*p* < 0.001) than the environmental variance, indicated that F_2_ families segregated for deterioration (data not shown). Broad sense heritability (*H*^2^) for the rate of deterioration ranged from 0.64 to 0.90. Because parents in all but one family differed in alleles at the SNP marker P-012.1-149 (“A” allele—slow deterioration, “G” allele—rapid deterioration) only this SNP was analyzed by KASP on individuals from six families. The plants from the remaining family (Bib × KH) were analyzed for alleles at the SNP marker P-012.1-096 using HRM approach. At this marker, the parent with slow deterioration (cv. King Henry) carries a “G” allele, while the rapidly deteriorating parent (cv. Bibb) carries an “A” allele. Genotyping with these two closely linked SNP markers revealed the same pattern in every family. The mean AUDePS of plants homozygous for alleles predicting a slow rate of deterioration was significantly lower (*p* < 0.05) than the AUDePS of heterozygous plants or plants having only the rapid deterioration alleles (*p* < 0.01). Data pooled across all seven families show the mean AUDePS values for the three groups as 175, 309, and 397, respectively (Table 3). The *d/a* ratio in six out of seven families ranged from -0.33 to 0.33, indicating mostly an additive effect of the alleles. The value of 0.62 was detected in the Lob × PI family, indicating a potential incomplete dominance of the “PI” allele for the rapid rate of deterioration.

**Table 3:** Rate of deterioration observed for parental lines and the plants from seven F_2_ families

F_2_ family and parental phenotypes	Rate of deterioration in parents and F_2_ families			*H* ^2^	Rate of deterioration in F_2_ plants with different allelic status^b^			Dominance to additivity ratio (*d/a*)
	Parent with slow deterioration	F_2_ progeny	Parent with rapid deterioration		Homozygous—favorable alleles	Heterozygous	Homozygous—unfavorable alleles	
Lob × PI (slow × rapid)	84^a^ (64–105)	366 (337–395)	310 (279–341)	0.81	187 (147–228)	395 (376–413)	443 (420–465)	0.62
LG × Sal (rapid × slow)	154 (133–175)	207 (173–242)	433 (419–447)	0.90	81 (40–121)	168 (125–211)	317 (284–350)	−0.33
LB × TS (rapid × slow)	154 (124–185)	321 (294–347)	404 (391–417)	0.69	207 (163–250)	340 (315–364)	407 (369–444)	0.33
Pav × Eru (rapid × slow)	269 (243–294)	377 (355–399)	446 (429–462)	0.64	259 (203–315)	377 (361–393)	463 (444–483)	0.15
Bib × KH (rapid × slow)	126 (98–155)	276 (247–305)	420 (400–439)	0.72	185 (150–219)	275 (243–306)	413 (382–444)	−0.21
LG x BLM (rapid × slow)	99 (75–124)	288 (256–320)	433 (419–447)	0.85	112 (61–163)	262 (223–302)	382 (364–400)	0.11
BB × LB (slow × rapid)	151 (125–179)	325 (292–358)	404 (391–417)	0.87	143 (46–239)	303 (265–340)	410 (383–437)	0.20
Pooled F_2_ families	148 (135–161)	309 (296–321)	408 (396–420)	0.78	175 (154–197)	309 (294–324)	397 (384–412)	0.20

*H*^2^ broad sense heritability, *d/a* dominance to additivity ratio that indicates the mode of inheritance. The value of 1 indicates that alleles for rapid deterioration are dominant, the value of 0 indicates that alleles for rapid deterioration have an additive effect, and the value of −1 indicates that alleles for rapid deterioration are recessive

^a^Mean values of the area under the deterioration progress steps (AUDePS), and 95% confidence interval of the mean. Lower AUDePS score indicates a slower rate of deterioration.

^b^Allelic status at the SNP marker P-012.1-149 (six families) or P-012.1-096 (Bib × KH family, only)

## Discussion

Compared to our previous experience^7,9,13,14^, cv. Salinas deteriorated in this study faster than expected, while cv. Lobjoits and PI 491224 deteriorated slower than expected. These minor differences, however, did not affect estimates of heritability and marker-trait association. Cv. Eruption was previously classified as having an intermediate rate of deterioration^7^. This classification was confirmed in the present study, where cv. Eruption deteriorated faster (AUDePS = 269) than other parents with the slow rate of deterioration (AUDePS from 84 to 154), but slower than parents with the rapid rate of deterioration (AUDePS from 310 to 446) (Table 3). However, because the rate of deterioration seen in cv. Eruption is still acceptable for fresh-cut processing, this cultivar was grouped with slow deteriorating accessions when selecting SNPs for marker-assisted selection. If the “Eruption” haplotype needs to be separated from haplotypes of other, even more slowly deteriorating accessions, an additional SNP from the BACX – E45/M48-162.87 region (either Lsat_1_v8_lg_4_284,212,432 or Lsat_1_v8_lg_4_284,212,475, SNPs P-012.1-028 and P-012.1-071 in the P-012.1 amplicon) can be used (Fig. 5).

Per-bag broad sense heritability (*H*^2^) in this study ranged from 0.61 to 0.90 in RIL and F_2_ families. These estimates are in the range of what we previously reported for the S88 × LB population (0.56–0.87)^9^. These heritability values indicate amenability of the trait to rapid genetic improvement. We previously detected *qSL4*, a locus linked to the rate of deterioration in the S88 × LB population that controlled a high proportion (40–74%) of the variation for deterioration^9^. Analysis of the Pav × Par mapping population revealed a QTL located in the same chromosomal region. This QTL explaining 42–44% of the total phenotypic variation of the trait (Table 2). Markers linked to *qSL4* co-segregated with the rate of deterioration in seven F_2_ families, indicating that alleles at this QTL may affect deterioration in diverse types of lettuce. Many of the rapid deteriorating accessions tested in this research are important sources of disease resistance^1,11–14,17,28,29^. The inability to evaluate for deterioration in early generations is a common impediment to using these disease-resistant accessions as parents in breeding. The availability of molecular markers to select slow deteriorating progeny from rapid×slow deterioration crosses may facilitate the use of disease resistant but rapid deteriorating parents in lettuce breeding. Four single dominant genes (*Dm4*, *Dm7*, *Dm11*, and *Dm44*)^30^ and one QTL (*qDm4.2*)^28^ for resistance to downy mildew (*Bremia lactucae* Regel) are located at the same chromosomal region as *qSL4*. At least one of the resistance genes (*Dm7*) is located directly at the BACX-E45/M48-162.87 region, only ~300 kb away from SNPs used in the shelf-life assay. This indicates a strong linkage between one or more resistance genes and the rate of salad deterioration. Marker-assisted selection can be used to break a linkage drag when developing new breeding lines with slow rate of deterioration and desirable combinations of resistance genes unless rapid deterioration is a pleotropic phenotype of a desirable resistance gene.

It was previously determined that at least two other QTLs located on LG1 and LG9 in addition to *qSL4* can play a role in the lettuce deterioration process^9^. We did not find, however, any significant or even suggestive QTL at these linkage groups when data from the Pav × Par mapping population were analyzed. In the future, we will analyze other lettuce populations, determine the existence of additional QTLs involved in the rate of deterioration, and study the stability of this trait when plants from different environments are processed for salad. In addition, the work is underway to identify the gene(s) determining *qSL4*. Though, the two SNPs identified for MAS are located in a non-coding region, the annotated genome sequence^19^ indicates that the BACX-E45/M48-162.87 QTL region contains over 50 genes. Several of the genes are predicted to encode proteins involved in disease resistance, organogenesis, morphogenesis, senescence, metal ion transport, isoprene synthesis, or several other functions. Cloning of this gene will allow a detailed study of the deterioration process in fresh-cut lettuce. Sequencing of the gene will also allow detection of the alleles associated with different rates of deterioration and development of a marker assay based on these alleles. MAS assay based on the alleles of the gene itself would be even more accurate in predicting the rate of deterioration than the SNPs identified in this study that was designed from the loci linked to the *qSL4* gene. Meantime, molecular marker assays based on SNPs P-012.1-096 and P-012.1-149 can be used to identify lettuce genotypes with a slow rate of deterioration after processing for salad. These markers accurately predicted the rate of deterioration in plants originating from seven F_2_ families and different types of lettuce. A combination of results obtained with these two molecular SNP markers with information about phenotypes of parents can be used to accurately select slow deteriorating genotypes in breeding populations, thus contributing to the development of lettuce cultivars with superior shelf-life.
